# Role of PI3K/Akt-Mediated Nrf2/HO-1 Signaling Pathway in Resveratrol Alleviation of Zearalenone-Induced Oxidative Stress and Apoptosis in TM4 Cells

**DOI:** 10.3390/toxins14110733

**Published:** 2022-10-26

**Authors:** Wenlin Xu, Hao Zheng, Youtian Fu, Yayi Gu, Hui Zou, Yan Yuan, Jianhong Gu, Zongping Liu, Jianchun Bian

**Affiliations:** 1 College of Veterinary Medicine, Yangzhou University, 12 Wenhui East Road, Yangzhou 225009, China; 2 Jiangsu Co-Innovation Center for Prevention and Control of Important Animal Infectious Diseases and Zoonoses, Yangzhou 225009, China; 3 Joint International Research Laboratory of Agriculture and Agri-Product Safety of the Ministry of Education of China, Yangzhou University, Yangzhou 225009, China

**Keywords:** resveratrol, zearalenone, TM4 cells, oxidative damage, ROS, cytotoxicity, PI3K/Akt signaling pathway, Nrf2/HO-1 signaling pathway

## Abstract

Zearalenone (ZEA) is a common mycotoxin that induces oxidative stress (OS) and affects the male reproductive system in animals. Resveratrol (RSV) has good antioxidant activity and can activate nuclear factor erythroid 2-related factor (Nrf2) to protect cells through the phosphatidylinositol 3-kinase (PI3K)/protein kinase B (Akt) signaling pathway. The objective of this study was to investigate the protective effect and the mechanism of RSV on OS and apoptosis in TM4 cells induced by ZEA. Prior to being exposed to ZEA, TM4 cells were pretreated with RSV or the PI3K/Akt inhibitor LY294002. Cell viability was measured by Cell Counting Kit-8 (CCK-8) assays. Flow cytometry was used to determine the level of apoptosis and intracellular reactive oxygen species (ROS). The expression of poly ADP-ribose polymerase (PARP), caspase-3, BCL2-associated X (Bax)/B-cell lymphoma-2 (Bcl-2), and PI3K/Akt-mediated Nrf2/heme oxygenase 1 (HO-1) signaling pathway-related proteins was evaluated by Western blotting. Nrf2 siRNA transfection and LY294002 treatment were used to investigate the role of the Nrf2/HO-1 and PI3K/Akt signaling pathways in RSV alleviation of ZEA-induced OS. The results showed that pretreatment with RSV significantly reduced the expression of apoptosis-related proteins and increased cell viability. Catalase (CAT) activity and glutathione (GSH) levels were also increased, whereas malondialdehyde (MDA) and ROS levels decreased (*p* < 0.05). RSV also upregulated Akt phosphorylation, Nrf2 nuclear translocation, and HO-1 expression under conditions of OS (*p* < 0.05). Transfection with Nrf2 siRNA abolished the protective effects of RSV against ZEA-induced cytotoxicity (*p* < 0.05), ROS accumulation (*p* < 0.05), and apoptosis (*p* < 0.05). LY294002 completely blocked the RSV-mediated increase in Nrf2 nuclear translocation (*p* < 0.05), HO-1 expression (*p* < 0.05), and cytoprotective activity (*p* < 0.05). Collectively, the above findings indicate that RSV can protect against ZEA-induced OS and apoptosis in TM4 cells by PI3K/Akt-mediated activation of the Nrf2/HO-1 signaling pathway.

## 1. Introduction

Zearalenone (ZEA) is a common nonsteroidal estrogenic mycotoxin present in grains and feed that can combine with estrogen receptors in cells of the body to exert estrogen-like effects [1]. ZEA can induce oxidative stress, DNA damage, mitochondrial damage, apoptosis, and autophagy [2,3,4,5]. ZEA has been shown to exert reproductive toxicity, genetic toxicity, and testicular toxicity in animals, as well as relatively high cytotoxicity, which can induce apoptosis in Sertoli cells [6,7]. Sertoli cells play an integral role in spermatogenesis and are potential targets for reproductive dysfunction induced by environmental poisons in male mice [8]. Oxidative stress (OS) is widely thought to result in damage to spermatogenic cells through the intrinsic mitochondrial apoptotic pathway, thereby leading to functional and metabolic abnormalities in male reproductive cells [9].

Nuclear factor erythroid 2-related factor 2 (Nrf2) is a key regulatory transcription factor involved in mediating the cellular antioxidant mechanism [10,11]. Nrf2 is normally located primarily in the cytoplasm, and it binds to Kelch-like ECH-associated protein 1 (Keap1), which is degraded by the proteasome pathway [12,13]. When cells are subjected to OS or electrophilic stimulation, Nrf2 dissociates from Keap1 and translocates to the nucleus where it binds to and induces the expression of antioxidant response elements (ARE) in the promoters of antioxidant genes to regulate the cellular antioxidant response [14].

Resveratrol (RSV) is a plant extract mainly existing in grapes, *Polygonum cuspidatum*, and peanuts [15]. It is an antitoxin with a variety of biological functions, including immune regulation, antioxidant, cardiovascular system protection, and tumor growth inhibition [16,17]. RSV has been shown to protect cells by directly decreasing the production of ROS or by indirectly upregulating the expression of cellular defense genes [18,19]. RSV can also enhance the activities of antioxidant enzymes and free-radical scavengers, thereby effectively alleviating OS [20,21]. RSV plays an important role in regulating cellular defense systems and protects cells from OS. It can increase the level of GSH and maintain the cellular redox balance by increasing the expression of HO-1, CAT, glutathione peroxidase (GSH-Px), superoxide dismutase (SOD), and other antioxidant defense enzymes [22].

RSV can activate Nrf2 through the PI3K/Akt signaling pathway to protect cells [14,23]. Activation of the PI3K/Akt signaling pathway can alleviate OS and cell injury [24], as well as inhibit autophagy and apoptosis [25]. Long et al. demonstrated that proanthocyanidin could protect against ZEA-induced oxidative damage and apoptosis in TM4 cells [26,27]. However, the mechanism underlying the protective effects of RSV are not fully understood, especially the role of the PI3K/Akt signaling pathway in protecting TM4 cells from ZEA-induced OS and apoptosis. TM4 cells represent the cell line culture derived from immature BALB/c mouse testis that possess major properties of normal Sertoli cells. Therefore, the purpose of this study was to investigate the protective effect of RSV on ZEA-induced OS and apoptosis in TM4 cells and its possible molecular mechanism so as to provide evidence in support of the use of RSV as a feed additive in livestock and poultry production.

## 2. Results

### 2.1. Effects of RSV on ZEA-Induced TM4 Cytotoxicity

**Figure 1:**
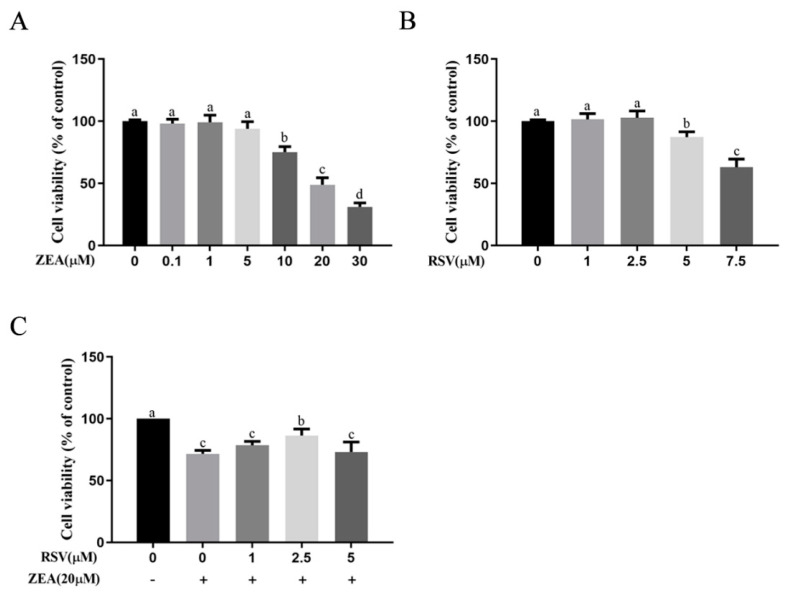
Effects of RSV on ZEA-induced TM4 cytotoxicity. TM4 cells were seeded into a 96-well plate, and the viability analysis of TM4 cells was measured by CCK-8 assay. (**A**) Effects of different concentrations of ZEA on TM4 cell viability. TM4 cells were treated with increasing concentrations of ZEA (0, 0.1, 1, 5, 10, 20, and 30 μmol·L^−1^) for 24 h. (**B**) Effects of different concentrations of RSV on TM4 cell viability. TM4 cells were treated with increasing concentrations of RSV (0, 1, 2.5, 5, and 7.5 μmol·L^−1^) for 24 h. (**C**) Effects of RSV on ZEA-induced TM4 cell viability. TM4 cells were exposed to ZEA (20 μmol·L^−1^) for 24 h after pretreatment with different concentrations of RSV for 3 h. Different letters (a, b, c, and d) represent a significant difference (*p* < 0.05 calculated by ANOVA for each group), while the same letters indicate no significant difference between groups.

To determine the most appropriate concentrations of ZEA and RSV for subsequent experiments, we treated TM4 cells with different concentrations of ZEA and RSV, and we then measured the viability of the treated cells using CCK-8 assays. As shown in Figure 1A, TM4 cells were incubated with ZEA at concentrations of 0, 0.1, 1, 5, 10, 20, and 30 μmol·L^−1^ for 24 h. These results showed that ZEA induced cell death in a dose-dependent manner. At 10 μmol·L^−1^ concentration and above, ZEA significantly reduced cell viability (*p* < 0.05). A high concentration of RSV exhibited a slight inhibitory effect on TM4 cell viability, and RSV significantly decreased the viability of TM4 cells at both 5 and 7.5 μmol·L^−1^ (*p* < 0.05) (Figure 1B). Preincubation of cells with RSV at 2.5 μmol·L^−1^ significantly attenuated the decrease in cell viability induced by ZEA (*p* < 0.05) (Figure 1C). On the basis of these results, 2.5 μmol·L^−1^ RSV and 20 μmol·L^−1^ ZEA were used in the subsequent experiments.

### 2.2. Effects of RSV on ZEA-Induced Apoptosis of TM4 Cells

**Figure 2:**
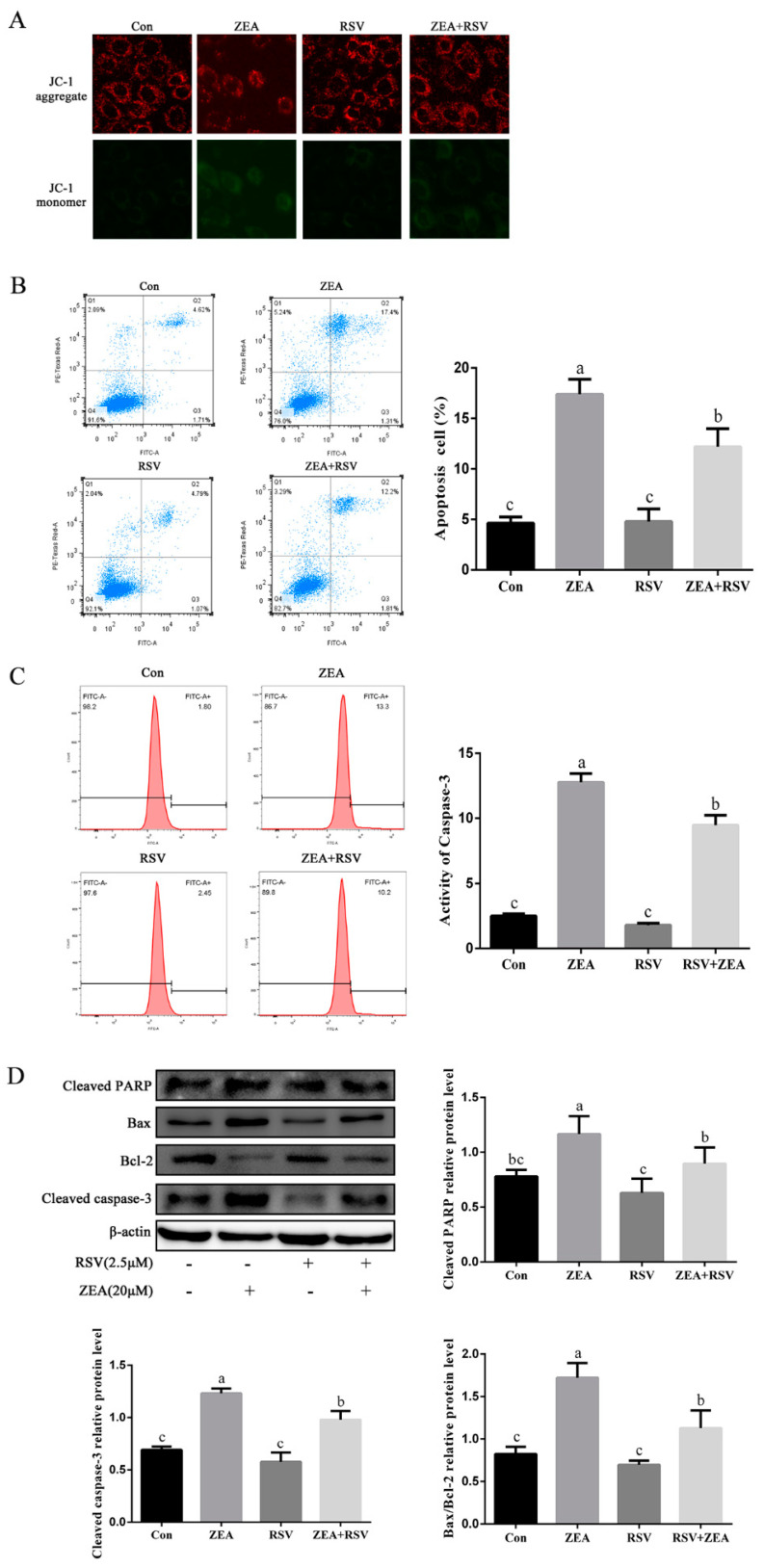
Effects of RSV on ZEA-induced apoptosis of TM4 cells. TM4 cells were seeded into six-well plates and exposed to ZEA (20 μmol·L^−1^) for 24 h after pretreatment with RSV (2.5 μmol·L^−1^) for 3 h. (**A**) JC-1 staining of TM4 cells (100× magnification). TM4 cells were incubated with JC-1 (10 μg/mL) for 30 min at 37 °C and the MMP levels were analyzed by flow cytometry. A higher ratio of green/red fluorescence indicates decreased MMP. (**B**) Flow cytometry analysis of TM4 cell apoptosis. Cells were collected and treated with 5 μL of Annexin V-FITC and 5 μL of PI, and then incubated in the dark for 20 min at 25 °C. (**C**) Activity of caspase-3 in TM4 cells was measured using an active caspase-3 staining kit and detected by flow cytometry. (**D**) The apoptosis-related protein levels of cleaved PARP, cleaved caspase-3, and Bax/Bcl-2 in TM4 cells. Different letters (a, b, c) represent a significant difference (*p* < 0.05 calculated by ANOVA for each group), while the same letters indicate no significant difference between groups.

The protective effect of RSV against the ZEA-induced reduction in mitochondrial membrane potential (MMP) in TM4 cells was investigated by measurement of JC-1 staining using fluorescence microscopy. A higher ratio of green/red fluorescence indicates decreased MMP. As shown in Figure 2A, the sizeable ZEA-induced increase in the green-to-red fluorescence ratio compared with the control group indicates decreased MMP after 24 h of ZEA treatment, and this increase was inhibited by pretreatment of the cells with RSV for 3 h (*p* < 0.05). Apoptosis of TM4 cells evaluated by flow cytometry showed that ZEA exposure significantly increased the number of apoptotic TM4 cells compared to the control group, whereas pretreatment with RSV attenuated TM4 cell apoptosis (*p* < 0.05) (Figure 2B). In addition, pretreatment with RSV significantly attenuated the increased caspase-3 activity in TM4 cells caused by exposure to ZEA (*p* < 0.05) (Figure 2C). As shown in Figure 2D, pretreatment with RSV significantly repressed the levels of apoptosis-related proteins such as cleaved PARP, cleaved caspase-3, and Bax/Bcl-2 (*p* < 0.05). These results indicated that 2.5 μmol·L^−1^ 3 h pretreatment with RSV stabilized the MMP and repressed ZEA-induced TM4 cell apoptosis.

### 2.3. Effects of RSV on ZEA-Induced Oxidative Stress in TM4 Cells

**Figure 3:**
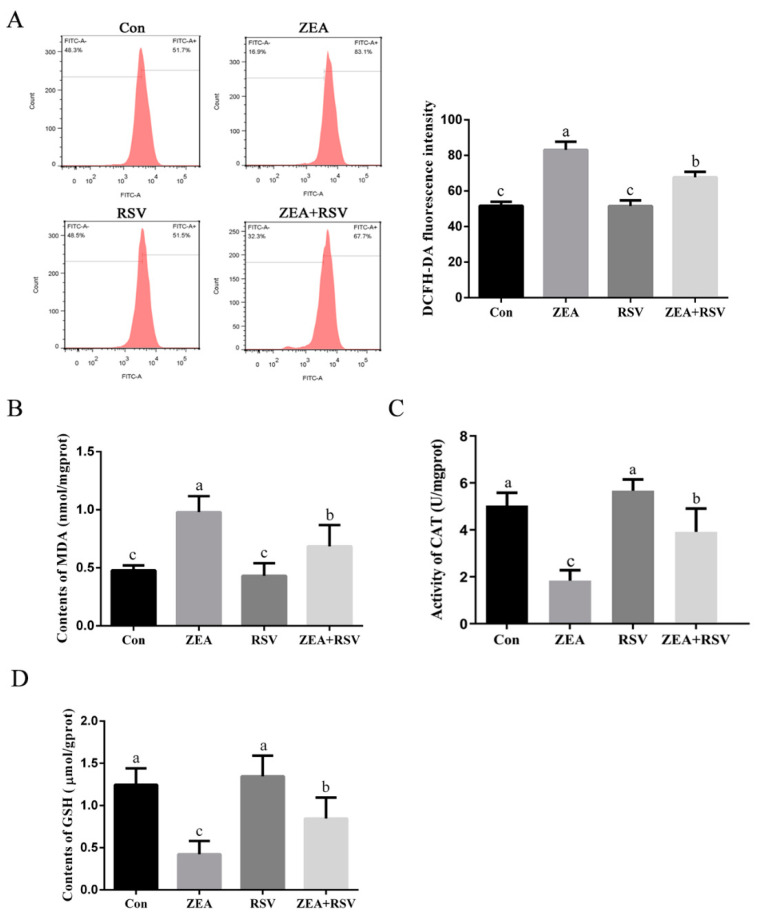
Effects of RSV on ZEA-induced oxidative stress in TM4 cells. TM4 cells were seeded into 60 mm plates and exposed to ZEA (20 μmol·L^−1^) for 24 h after pretreatment with RSV (2.5 μmol·L^−1^) for 3 h. (**A**) Intracellular ROS levels of TM4 cells. Cells were incubated with 10 μmol·L^−^^1^ DCFH-DA for 30 min at 37 °C in the dark. The ROS levels were analyzed by flow cytometry. (**B**) Contents of MDA in TM4 cells. (**C**) Activity of CAT in TM4 cells. (**D**) Contents of GSH in TM4 cells. Different letters (a, b, c) represent a significant difference (*p* < 0.05 calculated by ANOVA for each group), while the same letters indicate no significant difference between groups.

The effects of RSV on the levels of antioxidant markers and intracellular ROS in response to ZEA stimulation were assessed using specific assay kits. The fluorescence intensity can demonstrate the level of ROS. As shown in Figure 3A, the level of intracellular ROS was significantly increased by exposure of TM4 cells to ZEA, whereas pretreatment of cells with RSV decreased the accumulation of intracellular ROS induced by exposure to ZEA (*p* < 0.05). RSV significantly decreased the MDA content in ZEA-treated cells (*p* < 0.05) (Figure 3B). As shown in Figure 3C,D, exposure to ZEA markedly reduced CAT activity and GSH levels, and these effects were blocked by pretreatment with RSV (*p* < 0.05). These results indicate that RSV alleviates ZEA-induced OS in TM4 cells by inhibiting intracellular ROS accumulation and enhancing antioxidant enzyme activity.

### 2.4. Role of the Nrf2/HO-1 Signaling Pathway in Alleviation of ZEA-Induced TM4 Cell Injury by RSV

**Figure 4:**
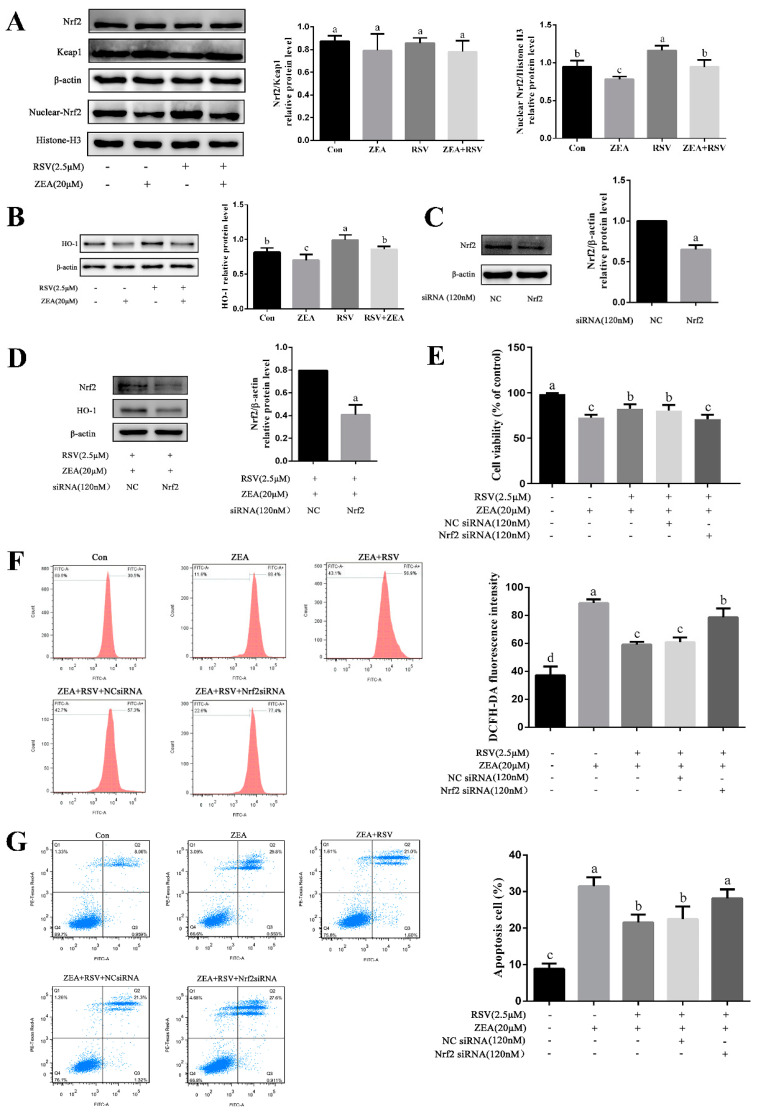
Role of the Nrf2/HO-1 signaling pathway in alleviation of ZEA-induced TM4 cell injury by RSV. TM4 cells were exposed to ZEA (20 μmol·L^−1^) for 24 h after pretreatment with RSV (2.5 μmol·L^−1^) for 3 h after transfection with NC siRNA or Nrf2 siRNA, using Lipofectamine™ 3000 for 12 h. (**A**) Expression levels of Nrf2/Keap1 and nuclear-Nrf2/Histone-H3 relative proteins (total protein and nuclear protein). (**B**) Expression levels of HO-1 relative proteins in TM4 cells. (**C**,**D**) Expression levels of Nrf2 and HO-1 relative proteins. (**E**) The viability of TM4 cells detected by CCK-8 assay. (**F**) Intracellular ROS levels of TM4 cells were assessed using a DCFH-DA ROS assay kit. The ROS levels were analyzed by flow cytometry. (**G**) Flow cytometry analysis of TM4 cell apoptosis was detected using an Annexin V-FITC/PI apoptosis kit. Different letters (a, b, c, d) represent a significant difference (*p* < 0.05 calculated by ANOVA (**A**,**B**,**E**–**G**) or a *t*-test (**C**,**D**) for each group), while the same letters indicate no significant difference between groups.

Western blot results (Figure 4A) showed that exposure to ZEA markedly inhibited the nuclear accumulation of Nrf2, but this response was blocked by pretreatment with RSV (*p* < 0.05). As shown in Figure 4B, ZEA exposure significantly decreased the protein expression of its downstream gene, HO-1, compared to that in the controls (*p* < 0.05). RSV pretreatment significantly attenuated the inhibitory effect of ZEA on HO-1 protein expression (*p* < 0.05). Moreover, to confirm the role of Nrf2 in protecting TM4 cells from ZEA-induced damage, cells were transfected with Nrf2 siRNA and then exposed to RSV and/or ZEA (*p* < 0.05). As shown in Figure 4C,D, treatment with Nrf2 siRNA reduced the relative protein expression of Nrf2 (*p* < 0.05). Transfection with Nrf2 siRNA significantly decreased the effect of RSV pretreatment on the ZEO-induced changes in the nuclear accumulation of Nrf2 and the HO-1 protein level (*p* < 0.05). The CCK-8 assay results showed that the protective effect of RSV on ZEA-induced apoptosis was eliminated after transfection with Nrf2 siRNA (*p* < 0.05) (Figure 4E). Flow cytometric analysis demonstrated that transfection with Nrf2 siRNA eliminated the reduction in intracellular ROS levels by pretreatment of cells with RSV prior to exposure to ZEA, as indicated by the increased fluorescence intensity (*p* < 0.05) (Figure 4F). Similarly, as shown in Figure 4G, the same experimental protocol showed that pretreatment with RSV significantly decreased the proportion of ZEA-induced apoptotic cells (*p* < 0.05). These results suggest that RSV alleviates ZEA-induced TM4 cell injury through the Nrf2/HO-1 signaling pathway.

### 2.5. Role of the PI3K/Akt Signaling Pathway in Alleviation of ZEA-Induced TM4 Cell Injury by RSV

**Figure 5:**
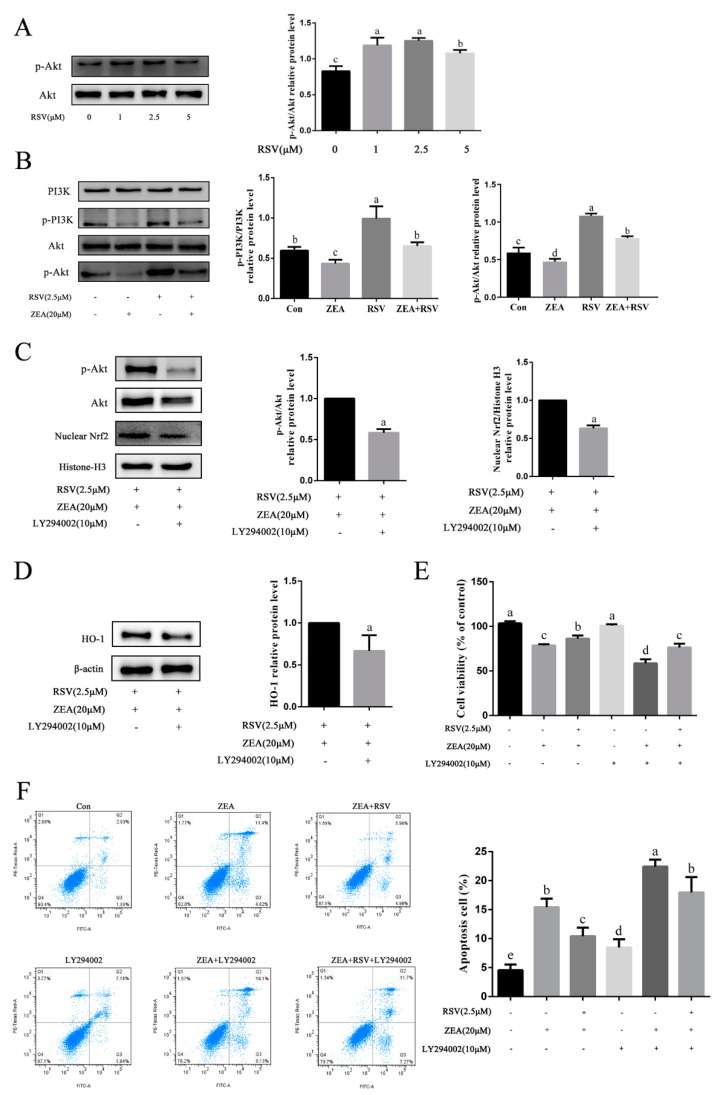
Role of the PI3K/Akt signaling pathway in alleviation of ZEA-induced TM4 cell injury by RSV. (**A**) Expression levels of p-Akt (ser 473) and Akt relative proteins in TM4 cells. TM4 cells were treated with different concentrations of RSV (0, 1, 2.5, and 5 μmol·L^−1^) for 24 h. (**B**) Expression levels of p-Akt (ser 473), p-PI3K, PI3K, and Akt relative proteins in TM4 cells. TM4 cells were pretreated with RSV (2.5 μmol·L^−1^) for 3 h, and then treated with or without ZEA (20 μmol·L^−1^) for 24 h. (**C**) Expression levels of p-Akt (ser 473), Akt, and Nrf2 nuclear accumulative relative proteins in TM4 cells. TM4 cells were incubated with LY294002 (10 μmol·L^−1^) for 1 h after pretreatment with RSV (2.5 μmol·L^−1^) for 3 h, then exposed to ZEA (20 μmol·L^−1^) for 24 h. (**D**) The HO-1 relative protein expression level in TM4 cells. (**E**) The viability of TM4 cells detected by CCK-8 assay. (**F**) Apoptosis of TM4 cells analyzed by flow cytometry. Cells were treated as described above and mixed with 5 μL of Annexin V-FITC and 5 μL of PI, and then incubated in the dark for 20 min at 25 °C. Different letters (a, b, c, d, e) represent a significant difference (*p* < 0.05 calculated by ANOVA (**A**,**B**,**E**,**F**) or a *t*-test (**C**,**D**) for each group), while the same letters indicate no significant difference between groups.

We also assessed whether RSV could alleviate OS through the PI3K/Akt signaling pathway. As shown in Figure 5A, Western blot results indicated that pretreatment with RSV significantly increased the protein expression of p-Akt (*p* < 0.05). Exposure to ZEA markedly inhibited p-PI3K and p-Akt levels, whereas these responses were reversed by pretreatment with RSV (*p* < 0.05) (Figure 5B). To examine the role of Akt in RSV-induced protection against ZEA-induced damage, we pretreated TM4 cells with the PI3K/Akt inhibitor LY294002 (10 μmol·L^−1^) for 1 h, followed by treatment of the cells with RSV and/or ZEA. Western blot results (Figure 5C) showed that LY294002 treatment significantly decreased the protein expression of p-Akt and nuclear accumulation of Nrf2 (*p* < 0.05). As shown in Figure 5D, the protein expression of HO-1 was also significantly decreased (*p* < 0.05). The CCK-8 assay results showed that LY294002 treatment significantly reduced the protective effect of RSV against ZEA-induced reduction in cell viability (*p* < 0.05) (Figure 5E). TM4 cells were labeled with Annexin V-FITC and PI, and apoptosis of cells was detected by flow cytometry. Flow cytometric analysis demonstrated that pretreatment with RSV significantly increased the proportion of ZEA-induced apoptotic cells, and this effect was eliminated by LY294002 treatment (*p* < 0.05) (Figure 5F). These results indicate that Akt activation is necessary for Nrf2 nuclear translocation and the cytoprotective effects induced by RSV.

## 3. Discussion

ZEA is one of the secondary metabolites produced by *Fusarium* fungi and can cause estrogen hyperactivity in animals [28]. The reproductive system is more sensitive to ZEA toxicity than other systems (gastrointestinal tract, lymphatic, inner organs, etc.) [29]. Exposure of animals to ZEA-contaminated feed adversely affects their reproductive ability [30]. In recent years, the reproductive toxicity of ZEA in male animals and its mechanisms of action have attracted extensive attention. Since ZEA can induce a variety of toxic mechanisms and pose a serious threat to humans and animals, an effective antidote is needed as an additive for animal food safety to protect animals from ZEA [31].

RSV is a natural polyphenolic compound present at high levels in a variety of plants and has been shown to have antioxidant effects and to act as an indirect antioxidant to induce the expression of antioxidant enzymes [32]. However, the mechanism via which RSV alleviates ZEA-induced OS and apoptosis in TM4 cells is not fully understood, especially the protective role of the PI3K/Akt signaling pathway in this process. To investigate the protective mechanism of RSV against ZEA-induced OS and cell apoptosis, cellular viability, the apoptosis rate, the MMP level, the expression of apoptosis-related proteins, and antioxidant and intracellular ROS levels were determined in TM4 cells. These results demonstrated that RSV had protective effects against ZEA-induced OS and apoptosis in TM4 cells. In addition, we found that the protective effect of RSV on TM4 cells involved activation of the Nrf2/HO-1 and PI3K/Akt signaling pathways.

It is widely thought that ZEA can induce OS in the testes, leading to mitochondrial dysfunction and germ cell apoptosis by the mitochondrial pathway [33,34,35]. Our study confirmed that ZEA could induce OS in TM4 cells, leading to cell apoptosis. ZEA seriously contaminates grain and feed, and the positive detection rate of maize feed is as high as 85% in some areas [36,37]. The occurrence of ZEA in feed and food can reach 3261 μg/kg [38], and the dose of ZEA (20 μmol·L^−^^1^) used in our study is easy for animals to ingest and cause poisoning through grain feed. RSV has been reported to have a variety of therapeutic benefits, including antiaging, antioxidant, anticancer, and cardiovascular protective effects [39]. There are hundreds of drugs, foods, and cosmetics containing RSV on the market today [40]. However, under the premise of ensuring safety, the rational application of RSV as an animal feed additive in animal production and the detection method of RSV in feed need to be further studied. The effects of RSV on cell viability and apoptosis were also investigated. Our study found that low concentrations (under 2.5 μmol·L^−1^) of RSV have no effect on germ cells, while high concentrations (over 7.5 μmol·L^−1^) of RSV cause cell damage. Therefore, the added dose of RSV should be low to prevent adverse effects on animals. Flow cytometry and Western blotting results showed that RSV (2.5 μmol·L^−1^) can alleviate ZEA-induced oxidative damage of TM4 cells, while having a good protective effect on cells.

OS refers to an imbalance between oxidation and antioxidation activity in the body that results in a high level of ROS. Low ROS levels play a role in regulating cell survival signals, whereas excessive ROS levels lead to oxidative damage [2]. Intracellular ROS can directly reflect the OS levels, and MDA is a commonly used OS marker [41]. Endogenous antioxidants are the first line of defense against OS, and changes in their levels can indirectly reflect the OS degree. A decrease in endogenous antioxidants exacerbates OS, while an increase in their levels helps to eliminate oxidative damage [42]. It has been reported that ZEA can induce ROS and MDA production in germ cells. RSV is a polyphenolic compound with a strong free-radical-scavenging effect, and it can, therefore, reduce cell damage caused by OS and lead to an antioxidative cellular environment [43]. Therefore, the intracellular ROS level and antioxidant enzyme activity were measured to explore the effect of RSV on ZEA-induced OS in cells. The results showed that ZEA could increase ROS levels, increase the MDA content, decrease the GSH content, and decrease the CAT activity in TM4 cells. RSV pretreatment could inhibit the production of intracellular ROS, increase the MDA level, and enhance the activity of antioxidant enzymes in TM4 cells. These results indicate that RSV results in antioxidant effects and that it can increase the activity of endogenous antioxidant enzymes, which in turn should exert a protective effect against ZEA-induced oxidation in TM4 cells.

Nrf2 is a transcription factor, and Nrf2 translocation can mediate transcription of the HO-1 gene and contribute to redox balancing [22]. As a phase II detoxifying enzyme, HO-1 plays a powerful role in mediating antioxidative and inhibition of inflammatory diseases [44,45]. Normally, Nrf2 binds to Keap1 in the cytoplasm; however, under oxidative conditions (ROS stimulation), Nrf2 dissociates from Keap1 and translocates to the nucleus, where it can bind to ARE, thereby activating downstream target genes and regulating the transcription of cytoprotective antioxidant genes (HO-1, SOD1, GCLC, and GCLM) [14]. RSV has been shown to increase the expression of Nrf2 and HO-1 proteins to enhance the antioxidant capacity and protect cells from OS [46]. Therefore, this study investigated the role of the Nrf2/HO-1 signaling pathway in the RSV-mediated alleviation of ZEA-induced oxidative stress and apoptosis in TM4 cells. The results showed that ZEA inhibited Nrf2 nuclear accumulation and HO-1-related protein expression in TM4 cells. Thus, RSV pretreatment alleviated ZEA-induced TM4 cell injury through the Nrf2/HO-1 signaling pathway. Additionally, Nrf2 siRNA transfection significantly decreased the expression levels of Nrf2 and HO-1 proteins and the viability of TM4 cells, and it antagonized the protective effects of RSV on ZEA-induced cytotoxicity, OS injury, and cell apoptosis. These results indicated that Nrf2 mediated the protective effect of RSV against ZEA-induced injury in TM4 cells. These results are similar to previous reports that RSV can protect cells from oxidative damage by promoting Nrf2 nuclear translocation and upregulation of the expression of antioxidant genes [23].

PI3K/Akt is a cell survival signaling pathway upstream of many protective pathways, and it increases cell survival [25]. The PI3K/Akt signaling pathway can promote cell proliferation, inhibit cell apoptosis, and mediate activation of the Nrf2/HO-1 signaling pathway [47]. Therefore, the role of the PI3K/Akt signaling pathway in RSV-mediated alleviation of ZEA-induced injury in TM4 cells was also investigated in this study. The results showed that ZEA-induced oxidative stress inhibited the expression of p-PI3K and p-Akt-related proteins in TM4 cells, while RSV significantly increased Akt phosphorylation and upregulated the levels of p-PI3K and p-Akt-related proteins in TM4 cells under conditions of oxidative stress. These results indicate that RSV can activate the PI3K/Akt signaling pathway to counteract ZEA-induced OS in TM4 cells and, thus, maintain cell viability. To further assess the protective mechanism of RSV in TM4 cells, the PI3K-specific inhibitor LY294002 was used [48]. The results showed that LY294002 treatment significantly reduced the protective effect of RSV against the ZEA-induced reduction in cell viability. The increase in apoptosis further proved that the PI3K/Akt signaling pathway is involved in the protective mechanism of RSV in cells. Several studies have demonstrated that the PI3K/Akt signaling pathway can enhance the Nrf2/HO-1 signaling pathway to protect cells [49,50]. Therefore, LY294002 was used to investigate the role of the PI3K/Akt signaling pathway in RSV in alleviating ZEA-induced reduction in HO-1 protein levels in TM4 cells. The results showed that LY294002 significantly downregulated HO-1 protein levels, thus indicating that the PI3K/Akt signaling pathway was involved in the activation of the Nrf2/HO-1 signaling pathway. Further studies are needed to improve the stability of RSV as a feed additive and the application of its derivatives.

## 4. Conclusions

**Figure 6:**
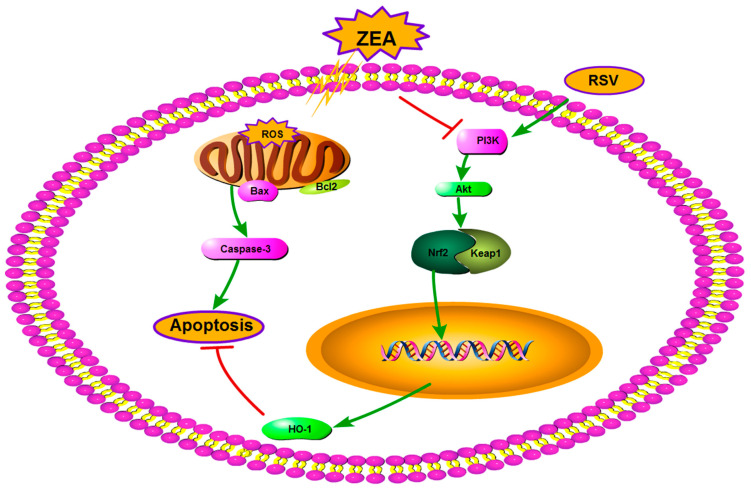
A proposed signaling pathway involved in RSV against ZEA-induced oxidative stress (OS) and apoptosis in TM4 cells. The schematic diagram shows that RSV induces Nrf2-mediated cytoprotective protein via activation of the PI3K/Akt signaling pathway, which protects against OS of TM4 cells. ZEA induces ROS generation results in cell apoptosis. Meanwhile, RSV activates Nrf2/HO-1 through the PI3K/Akt pathway. Activation of Nrf2/HO-1 protects against ZEA-induced apoptosis. Green arrows indicate stimulation, and red bars indicate inhibition.

In conclusion, this study demonstrated that ZEA can increase intracellular ROS levels, caspase-3 activity, and the expression of apoptosis-related proteins, as well as induce oxidative stress and apoptosis in TM4 cells. RSV stabilized the MMP and alleviated ZEA-induced TM4 cytotoxicity. In addition, RSV alleviated ZEA-induced OS in TM4 cells by activating the PI3K/Akt signaling pathway and inducing Nrf2-mediated cytoprotective protein expression. Thus, RSV inhibited ZEA-induced OS and apoptosis in TM4 cells by activating the Nrf2/HO-1 signaling pathway through the PI3K/Akt signaling pathway (Figure 6).

## 5. Materials and Methods

### 5.1. Chemicals and Reagents

Zearalenone (ZEA) and LY294002 were purchased from Sigma-Aldrich (St. Louis, MO, USA). Resveratrol (RSV) was purchased from Solarbio (Cat # SR8070; Beijing, China). Dulbecco’s modified Eagle medium/nutrient mixture F-12 (DMEM/F-12) was obtained from Gibco (Cat # 12500-062; Grand Island, NY, USA). Fetal bovine serum (FBS) was obtained from GeminiBio (Cat # 900-108; Sacramento, CA, USA). Radioimmunoprecipitation assay (RIPA) lysis buffer and protease inhibitor complex were purchased from Pulilai (Cat # C1053; Beijing, China). Lipofectamine™ 3000 was purchased from Thermo Fisher Scientific (Cat # L3000015; Carlsbad, CA, USA). Phenylmethanesulfonylfluoride (PMSF), JC-1, Cell Counting Kit-8 (CCK-8), bicinchoninic acid (BCA), and the dichlorofluorescein diacetate (DCFH-DA) ROS assay kit were purchased from Beyotime Institute of Biotechnology (Shanghai, China). The Annexin V/fluorescein isothiocyanate/propidium iodide (Annexin V-FITC/PI) apoptosis kit and FITC active caspase-3 apoptosis kit were obtained from BD Biosciences (San Jose, CA, USA). CAT, MDA, and GSH assay kits were purchased from the Jiancheng Bioengineering Institute (Nanjing, China). PI3K, p-PI3K, p-Akt, Bax, Bcl2, caspase-3, HO-1, and β-actin antibodies were purchased from Cell Signaling Technology (Danvers, MA, USA). c-PARP (ab32064), Nrf2 (ab137550), and histone H3 (ab1791) antibodies were purchased from Abcam (Cambridge, UK). All other reagents and chemicals were of analytical grade and were obtained commercially.

### 5.2. Cell Culture and Treatment

The Sertoli cell line TM4 was obtained from the American Type Culture Collection (ATCC, Rockefeller, MD, USA). Cells were seeded into plates, flasks, or dishes in DMEM/F-12 with high glucose supplemented with 10% FBS, 0.5% penicillin/streptomycin 10,000 U·mL^−1^ (Gibco Life Technologies, Grand Island, NY, USA), and cultured at 37 °C with 5% CO_2_. TM4 cells were seeded when the cells reached 80% and treated with ZEA (0, 0.1, 1, 5, 10, 20, and 30 μmol·L^−1^) or RSV (0, 1, 2.5, and 5 μmol·L^−1^) for 24 h. The effect of RSV on the ZEA-induced reduction in cell proliferation was measured by pretreating cells with different concentrations of RSV (0, 1, 2.5, and 5 μmol·L^−1^) for 3 h, and then treating these cells with ZEA (20 μmol·L^−1^) for 24 h. The mechanism of the protective effect was evaluated by pretreatment with LY294002 and ZEA-treated cells (10 μmol·L^−1^ LY294002 treated for 1 h and 20 μmol·L^−1^ ZEA treated for 24 h) with or without RSV for 3 h.

### 5.3. Measurement of Cell Viability by CCK-8 Assay

Cell Counting Kit-8 (CCK-8) was used to assay TM4 cell proliferation. The cells were detached with 0.25% trypsin and washed with culture medium to deactivate the trypsin, and then the cell suspension was collected. Briefly, cells were seeded into individual 96-well plates at a density of 5 × 10^4^·mL^−1^, 100 μL per well, and incubated under the above conditions. After 24 h, CCK-8 solution (10 μmol·L^−1^) was added to each well, and the absorbance was measured using an ELx800 Absorbance Microplate Reader (Bio Tek Instruments, Winooski, VT, USA) at 450 nm after incubation for 1 h in an incubator.

### 5.4. Apoptosis Detection by Flow Cytometry

Apoptosis of TM4 cells was detected using an Annexin V-FITC/PI apoptosis kit. After treatment as described above, TM4 cells were washed twice with precooled PBS, and then centrifuged at 300× *g* for 5 min at 4 °C for each time. The cells were collected at a density of 5 × 10^5^·mL^−1^ in a six-well plate and treated with 5 μL of Annexin V-FITC and 5 μL of PI after adding 100 μL of 1× Binding Buffer to each well. The cells were then incubated in the dark for 20 min at room temperature (25 °C), and the reaction was terminated by adding 400 μL of 1× Binding Buffer. Apoptosis was detected by flow cytometry (FACS LSRFortessa, BD Biosciences, San Jose, CA, USA) within 1 h of adding the reagent. The data were analyzed using FlowJo™ software (version 2.0) (BD Biosciences, San Jose, CA, USA).

### 5.5. Measurement of the Mitochondrial Membrane Potential (MMP)

The mitochondrial membrane potential (MMP) was assayed by JC-1 staining and flow cytometry. In brief, TM4 cells were treated according to the cell treatment above, and the cells were then harvested, washed with PBS, and incubated with JC-1 (10 μg/mL) for 30 min at 37 °C. The MMP levels were analyzed by flow cytometry (FACS LSRFortessa, BD Biosciences, San Jose, CA, USA).

### 5.6. Detection of Intracellular ROS by Flow Cytometry

ROS levels were assessed using a DCFH-DA ROS assay kit. TM4 cells were seeded in six-well plates and treated according to the cell treatment above. After treatment, the cells were harvested, washed, and incubated with 10 μmol·L^−1^ DCFH-DA for 30 min at 37 °C in the dark. The ROS levels were analyzed by flow cytometry (FACS LSRFortessa, BD Biosciences, San Jose, CA, USA).

### 5.7. Measurement of GSH CAT MDA

Cellular OS levels were assayed by measuring the levels of antioxidant markers such as MDA and GSH. The activity of MDA and GSH, and the contents of CAT in TM4 cells were measured using MDA, GSH, and CAT assay kits according to the manufacturer’s instructions.

### 5.8. siRNA Transfection

TM4 cells were seeded in six-well plates for 12 h and then transfected with control siRNA (NC siRNA) or Nrf2 siRNA (target sequence 5′-GCACAATGGAATTCAATGA-3′) synthesized by Ribobio Co. Ltd. (Guangzhou, China), using Lipofectamine™ 3000 for 12 h. After transfection, the TM4 cells were incubated with RSV for 3 h before ZEA treatment. The protective effect of RSV was evaluated by Western blotting or CCK-8 assay.

### 5.9. Western Blot Analysis

TM4 cells were placed on ice, the supernatant was discarded, the cells were washed twice with PBS, and the remaining liquid was removed by wicking with filter paper. A 100 μL aliquot of RIPA lysis buffer was added for lysis on ice for 5 min. The cells were evenly scraped into tubes with a cell scraper, lysed at 4 °C for 30 min, and then sonicated for 30 s. After centrifugation at 10,000× *g* for 10 min at 4 °C, the supernatant was collected. A BCA protein concentration assay kit was used to detect the protein concentration, and the protein concentration of each group was adjusted to be consistent. The protein samples were mixed with SDS loading buffer, which were separated by polyacrylamide gel electrophoresis (PAGE). After electrophoresis, the proteins were transferred to polyvinylidene difluoride (PVDF) membranes and blocked with TBST containing 5% skim milk for 1.5 h at room temperature. Primary antibodies were diluted (1:1000) in TBST containing 5% skim milk. The PVDF membrane strips were incubated with specific primary antibodies (monoclonal anti-HO-1, Nrf2, PI3K, Akt, Bcl-2, Bax, caspase-3, etc.) overnight at 4 °C. After washing with TBST five times, the strips were incubated with the corresponding horseradish peroxidase (HRP)-conjugated secondary antibody (1:10,000) for 2 h at room temperature (25 °C). After washing with TBST again, the antigens were detected by enhanced chemiluminescence (NCM Biotech, Suzhou, China). Densitometry was analyzed using ImageJ software (version 1.8.0) (National Institute of Health, Bethesda, MD, USA), and quantitative analysis was performed using β-actin as a loading control.

### 5.10. Statistical Analysis

Analysis of variance (ANOVA) or a *t*-test was used to analyze the differences between groups. All data are expressed as the mean ± SEM, and a value of *p* < 0.05 was considered statistically significant. All statistical data were analyzed using GraphPad Prism software (version 6.0).

## Data Availability

Not applicable.
